# Efficient and accurate determination of genome-wide DNA methylation patterns in *Arabidopsis thaliana* with enzymatic methyl sequencing

**DOI:** 10.1186/s13072-020-00361-9

**Published:** 2020-10-07

**Authors:** Suhua Feng, Zhenhui Zhong, Ming Wang, Steven E. Jacobsen

**Affiliations:** 1 Department of Molecular, Cell and Developmental Biology, University of California at Los Angeles, Los Angeles, CA 90095 USA; 2 Eli and Edythe Broad Center of Regenerative Medicine and Stem Cell Research, University of California at Los Angeles, Los Angeles, CA 90095 USA; 3 Howard Hughes Medical Institute, University of California at Los Angeles, Los Angeles, CA 90095 USA

**Keywords:** DNA methylation, Bisulfite sequencing, WGBS, EM-seq, TET, APOBEC, Arabidopsis flowers, Arabidopsis leaves

## Abstract

**Background:**

5′ methylation of cytosines in DNA molecules is an important epigenetic mark in eukaryotes. Bisulfite sequencing is the gold standard of DNA methylation detection, and whole-genome bisulfite sequencing (WGBS) has been widely used to detect methylation at single-nucleotide resolution on a genome-wide scale. However, sodium bisulfite is known to severely degrade DNA, which, in combination with biases introduced during PCR amplification, leads to unbalanced base representation in the final sequencing libraries. Enzymatic conversion of unmethylated cytosines to uracils can achieve the same end product for sequencing as does bisulfite treatment and does not affect the integrity of the DNA; enzymatic methylation sequencing may, thus, provide advantages over bisulfite sequencing.

**Results:**

Using an enzymatic methyl-seq (EM-seq) technique to selectively deaminate unmethylated cytosines to uracils, we generated and sequenced libraries based on different amounts of Arabidopsis input DNA and different numbers of PCR cycles, and compared these data to results from traditional whole-genome bisulfite sequencing. We found that EM-seq libraries were more consistent between replicates and had higher mapping and lower duplication rates, lower background noise, higher average coverage, and higher coverage of total cytosines. Differential methylation region (DMR) analysis showed that WGBS tended to over-estimate methylation levels especially in CHG and CHH contexts, whereas EM-seq detected higher CG methylation levels in certain highly methylated areas. These phenomena can be mostly explained by a correlation of WGBS methylation estimation with GC content and methylated cytosine density. We used EM-seq to compare methylation between leaves and flowers, and found that CHG methylation level is greatly elevated in flowers, especially in pericentromeric regions.

**Conclusion:**

We suggest that EM-seq is a more accurate and reliable approach than WGBS to detect methylation. Compared to WGBS, the results of EM-seq are less affected by differences in library preparation conditions or by the skewed base composition in the converted DNA. It may therefore be more desirable to use EM-seq in methylation studies.

## Introduction

The fifth carbon position of cytosine in DNA can be covalently modified by the addition of a methyl group to form 5-methylcytosine (5-mC). DNA methylation that takes place at cytosine residues which are followed by guanine is termed CG methylation and is conserved in most eukaryotes. Non-CG methylation, where modification occurs in CHG and CHH contexts (where H corresponds to A, T, or C residues), occurs in plants and many other organisms. CHH methylation is also called asymmetrical methylation. DNA methylation is typically associated with the silencing of genes and repetitive DNAs such as transposable elements; however, expressed genes are also found to be methylated. DNA methylation is involved in a large number of cellular processes including genomic imprinting, X chromosome inactivation, embryonic development, and transcriptional regulation of developmentally important genes, as well as in ensuring genome integrity and protecting against invasive DNAs [1–5].

The first step in the study of DNA methylation is to determine whether or not a given cytosine residue is methylated. Indirect approaches to measure methylation include pull-down assays with methylation-specific antibodies and methyl-binding proteins, and restriction digestion with enzymes with preferences for or against methylcytosines [6]. The direct approach, which can achieve single-nucleotide resolution, is sequencing. Since methylated cytosine pairs with guanine in the same way unmethylated cytosine does, traditional sequencing methods (based on base-pairing) are not able to differentiate between methylated and unmethylated cytosines. To solve this problem, sodium bisulfite can be used to convert unmethylated cytosines to uracils, which are amplified as thymines in PCR; because methylated cytosines do not react with sodium bisulfite, they remain as cytosines in the sequence. Thus, thymines detected in bisulfite sequencing correspond to either thymines or unmethylated cytosines in the original DNA, and alignment with the original template sequence easily differentiates between them [7]. Since its development a little more than a decade ago, whole-genome bisulfite sequencing (WGBS) has been successfully used to survey DNA methylation on a genome-wide scale [8–11]. While WGBS (which combines bisulfite treatment with high-throughput sequencing) is the gold standard for measuring genome-wide methylation, it has several shortcomings. After bisulfite conversion, DNA becomes C-poor, which can result in difficulties for polymerase reactions, as well as with sequencing machines, basecallers, and aligners. Although recent improvements in PCR reagents, sequencing hardware/software, and bioinformatics tools have helped to alleviate these difficulties, two fundamental problems remain. First, bisulfite degrades the majority of the DNA during the conversion process (due to backbone scission induced by depyrimidination and perhaps depurination as well); second, bisulfite preferentially damages DNA at unmethylated cytosines via depyrimidination (more effectively than at methylated cytosines) [12, 13]. These properties of bisulfite treatment make it challenging to perform WGBS from tissues that have limited starting material, and create a bias that can result in an over-estimation of methylation level by WGBS.

In addition to chemicals like bisulfite, DNA bases can also react with enzymes. For example, 5-mC can be converted to 5-hydroxymethylcytosine (5-hmC), then to 5-formylcytosine (5-fC), and finally to 5-carboxylcytosine (5-caC) in a cascade of reactions regulated by the ten-eleven translocation (TET) family of dioxygenases [14, 15]. Both methylated and unmethylated cytosines can be deaminated by the apolipoprotein B mRNA editing enzyme catalytic polypeptide-like 3A (APOBEC3A) to generate thymines and uracils, respectively [16–18]. Interestingly, APOBEC3A has only negligible cytidine deaminase activity toward the TET-oxidized methylcytosines, 5-hmC, 5-fC, and 5-caC [19]. This creates an opportunity to use a combination of TET and APOBEC3A to differentiate between methylated and unmethylated cytosines.

Recently, an enzymatic methyl-seq (EM-seq) technique was developed, which uses TET2 in the first enzymatic step to oxidize methylated cytosines and APOBEC2 in the second enzymatic step to convert unmethylated cytosines to uracils [20]. During the subsequent PCR amplification, oxidized methylcytosines form base pairs with guanines and uracils form base pairs with adenines. Since the end products of WGBS and EM-seq are the same (methylated cytosines stay as cytosines and unmethylated cytosines appear as thymines in the sequence), the same analysis tools can be used. Because enzymatic reactions are non-destructive, EM-seq promises better yield and higher accuracy in the measurement of methylation levels [20]. In this study, we employed EM-seq to study DNA methylation in *Arabidopsis thaliana* and compared EM-seq results to those from WGBS. We also used EM-seq to examine the methylation differences between flowers and leaves in Arabidopsis.

## Results

### Generating and sequencing EM-seq and WGBS libraries

**Fig. 1:**
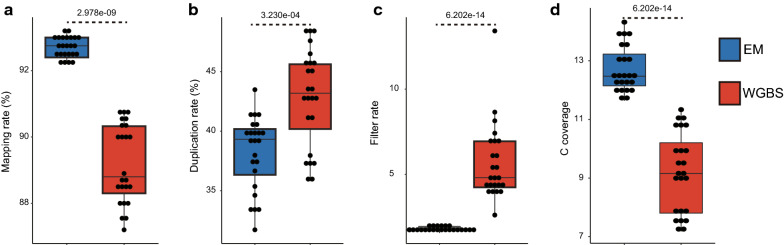
Quality comparison between EM-seq and WGBS. Boxplots showing: **a** mapping rates of EM-seq and WGBS. **b** duplication rates of EM-seq and WGBS. **c** read filtering rates of EM-seq and WGBS. Reads with three or more consecutive methylated CHH sites are considered as non-converted reads and removed from subsequent analyses. **d** Boxplot showing average coverages of methylated cytosines in EM-seq and WGBS. In **a**–**d**, blue box represents EM-seq and red box represents WGBS. The *P* values indicated at the top of each plot were estimated using a Student’s *t*-test

To systematically compare EM-seq and WGBS, we prepared Illumina sequencing libraries from different amounts of genomic DNA extracted from Arabidopsis flowers—25 ng, 50 ng, 150 ng, or 400 ng (to encompass the range of starting material amounts that are typically used in an Arabidopsis methylome study). This was followed by either enzymatic (TET2 followed by APOBEC3A) or bisulfite treatment, and PCR amplification of the converted products (for 6, 12, or 18 cycles) to complete the library preparations (Additional file 1: Fig. S1, see also Methods). Two replicates were performed for each condition. EM-seq libraries consistently showed higher mapping rates and lower duplication rates than WGBS libraries, and the variation between EM-seq libraries was smaller than the variation between WGBS libraries (Fig. 1ab, Additional file 2: Table S1a). In addition, within WGBS libraries, higher mapping rates and lower duplication rates (manifested by higher effective read rate) were generally associated with moderate input amounts and lower PCR cycle numbers (Additional file 2: Table S1a). One of the causes of false methylation reporting in bisulfite sequencing is non-conversion, in which the two strands of DNA occasionally fail to fully denature during bisulfite treatment and are thus resistant to bisulfite conversion [21]; this leads to the detection of several adjacent un-converted cytosines. In Arabidopsis, we previously introduced a filter that discards sequencing reads with three or more consecutive methylated cytosines in the CHH context [8]. This non-conversion filter works well in Arabidopsis, since CHHs are rarely methylated above 10% [22, 23], so the chance of observing three consecutive methylated CHHs is below 0.1% and only a small number of real methylation signatures are discarded. However, it is not practical to use this filter in organisms that have high levels of CHH methylation. Very few EM-seq reads (1.56%–2.01%) eligible for removal by the non-conversion filter were identified, while the filtered rates for WGBS libraries were much higher and showed greater variation (2.62% to 13.41%) (Fig. 1c, Additional file 2: Table S1b). This suggests that the EM-seq method is generally free of the notorious non-conversion problem that is frequently observed in WGBS, and is, therefore, useful for methylation detection in organisms in which use of an arbitrary non-conversion filter is not suitable. Consistent with this, we found that virtually no methylated cytosines were detected from the unmethylated Arabidopsis chloroplast genomes in any EM-seq library, a result that is only achieved or approached by the best WGBS library (400 ng input with 12 cycles of PCR) (Additional file 1: Fig. S2, Additional file 2: Table S1c). This indicates that EM-seq has much lower background noise levels than WGBS.

**Fig. 2:**
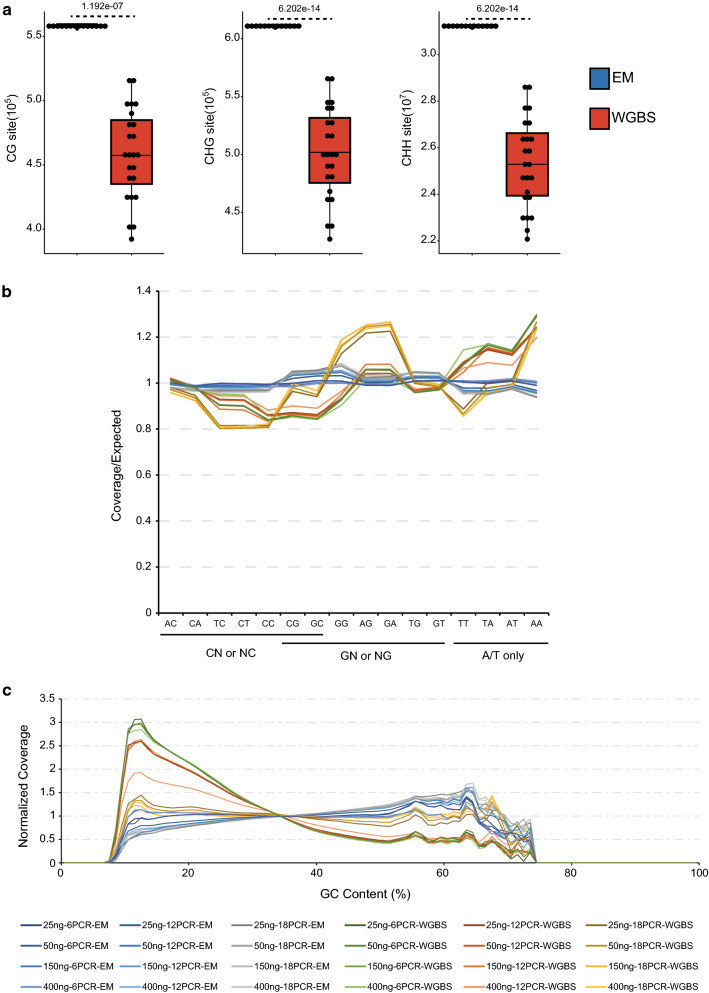
Coverage comparison between EM-seq and WGBS. **a** Boxplots showing numbers of methylated CG, CHG, and CHH cytosines identified by EM-seq and WGBS. Blue box represents EM-seq and red box represents WGBS. The *P* values shown at the top were estimated using a Student’s t-test. **b** Average coverage levels of different dinucleotides in EM-seq and WGBS. **c** Average coverages of genome-wide 400 bp bins, ranked by GC content, in EM-seq and WGBS. In **b** and **c**, coverage levels are normalized with expected coverages (total reads over reference genome). Colored lines represent different library preparations

As expected from these comparisons, EM-seq also displays advantages in terms of genomic coverage (Fig. 1d). Perhaps more importantly, EM-seq is able to cover 22.07%, 22.10%, and 23.47% more CG, CHG, and CHH sites, respectively, than WGBS (Fig. 2a); these numbers are consistent with the previous findings of EM-seq in human cells [20] and the manufacturer’s description of the product [24]. All EM-seq libraries exhibit similar cytosine coverage (CG: ranging from 5,556,957 to 5,602,669, CHG: ranging from 6,090,541 to 6,128,646, CHH: ranging from 31,123,001 to 31,315,262), while different preparation conditions clearly affect the performance of WGBS libraries (CG: ranging from 3,922,759 to 5,165,506, CHG: ranging from 4,26,9441 to 5,664,610, CHH: ranging from 22,080,267 to 28,678,168) (Fig. 2a).

Next, we examined the dependence of coverage on nucleotide composition. Dinucleotide profiles suggest that EM-seq has even coverage and is minimally affected by different dinucleotide combinations. In contrast, WGBS libraries show enrichment for dinucleotides containing Gs and depletion for dinucleotides contains Cs (Fig. 2b), consistent with the damaging effect of sodium bisulfite on unmethylated cytosines [13]. These biases are more pronounced in libraries with 18 cycles of PCR (Fig. 2b), indicating that PCR amplification during WGBS library preparation favors unconverted DNA over converted DNA, due to the low melting temperature and thermostability of AT pairs [25, 26]. This can further negatively affect accurate estimation of methylation level (see below). Similar biases were not observed in EM-seq libraries, although the product of TET2/APOBEC3A conversion is the same as that of bisulfite conversion, which suggests that the polymerase used to amplify EM-seq libraries is superior to the one used for WGBS, in terms of avoiding the introduction of base biases. As previously shown for a library preparation kit similar to the one used in our study [27], WGBS libraries show enrichment for dinucleotides containing only A or T (Fig. 2b). We then decided to look directly at the dependence of coverage on GC content. Again, EM-seq libraries show more even profiles over the majority of the GC content range than do WGBS libraries (Fig. 2c). WGBS libraries have clearly higher coverage in AT-rich regions than in GC-rich regions (Fig. 2c), a known issue for bisulfite sequencing [20, 27] that is discussed above (Fig. 2b).

Overall, the quality metrics of our EM-seq libraries encouraged us to further explore whether it measures methylation more accurately than WGBS in Arabidopsis.

### Arabidopsis DNA methylation levels measured by EM-seq and WGBS

**Fig. 3:**
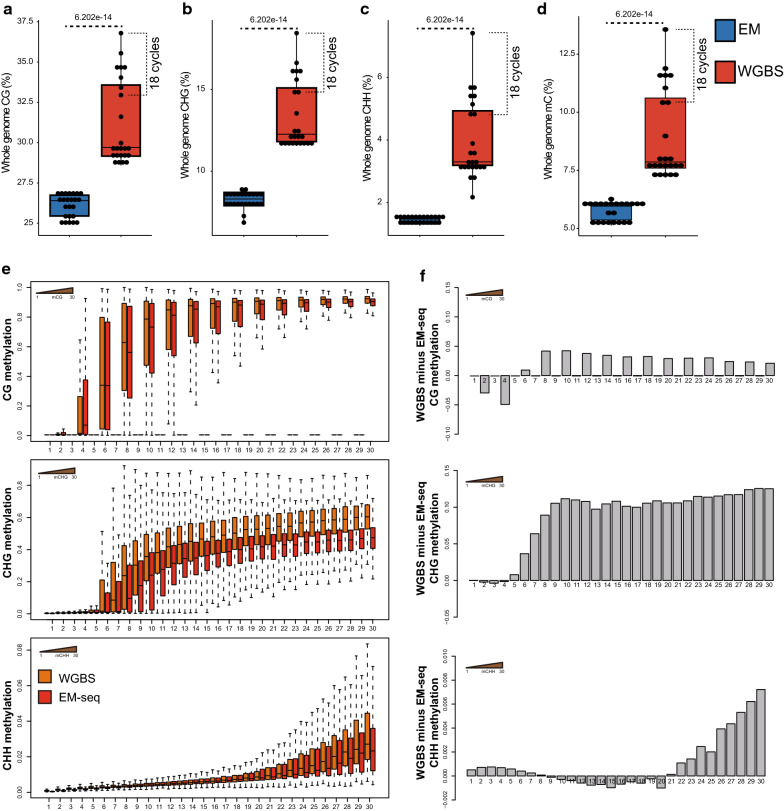
Comparison of methylation levels between EM-seq and WGBS. **a–d** Boxplots showing whole-genome CG (**a**), CHG (**b**), CHH (**c**), and C (**d**) methylation levels in EM-seq and WGBS. WGBS libraries prepared with 18 PCR cycles are indicated with brackets in the boxplots. Blue boxes represent EM-seq and red boxes represent WGBS. The *P* values, shown at the top of the plots, were estimated using a Student’s *t*-test. **e** Boxplots showing the relationship between methylated CG, methylated CHG, and methylated CHH counts (*x*-axis) and methylation levels (*y*-axis) in WGBS and EM-seq. **f** Methylation differences (average WGBS methylation levels minus average EM-seq methylation levels) from **e**. In **e** and **f**, for CG methylation, only regions with even numbers of CG sites have been analyzed, since CG methylation is symmetrical

We compared levels of CG, CHG, and CHH methylation measured by EM-seq and by WGBS and noted that EM-seq-measured DNA methylation levels are lower (Fig. 3a–c, Additional file 3: Table S2), even if background noise is considered (see Additional file 1: Fig. S2). This is consistent with the previous results obtained by the manufacturer for Arabidopsis [24]. Total DNA methylation levels estimated from EM-seq data (Fig. 3d) are close to the levels previously detected by liquid chromatography–mass spectrometry (LC–MS) in Arabidopsis [24]. We observed that increasing the number of PCR cycles led to higher methylation levels in the respective WGBS libraries (especially for libraries with 18 cycles of PCR; Fig. 3a–d, Additional file 3: Table S2) and reasoned that this likely reflects the above-mentioned preference of PCR amplification during WGBS library preparation for unconverted DNA (see Fig. 2b). To further test this hypothesis, we analyzed the correlation of methylation level with density of methylated cytosines in both EM-seq and WGBS. For this analysis, we picked the best WGBS library (400 ng input with 12 cycles of PCR) (see Additional file 1: Fig. S2, Additional file 2: Table S1c) and its EM-seq counterpart. As Fig. 3ef reveals, for CHG and CHH methylations, the differences in methylation levels between EM-seq and WGBS increases with cytosine methylation density, which is the expected result based on our hypothesis. Much less difference between EM-seq and WGBS was observed for CG methylation, possibly because CG methylation is more or less bimodal (either completely unmethylated or completely methylated) [8], and thus, the PCR bias in WGBS library preparation toward methylated CG sites would have less influence on CG methylation percentages. Nonetheless, CG methylation levels still appear to be moderately over-estimated by WGBS due to the selective damage of DNA containing unmethylated cytosines by sodium bisulfite [13] (see also Fig. 2bc).

**Fig. 4:**
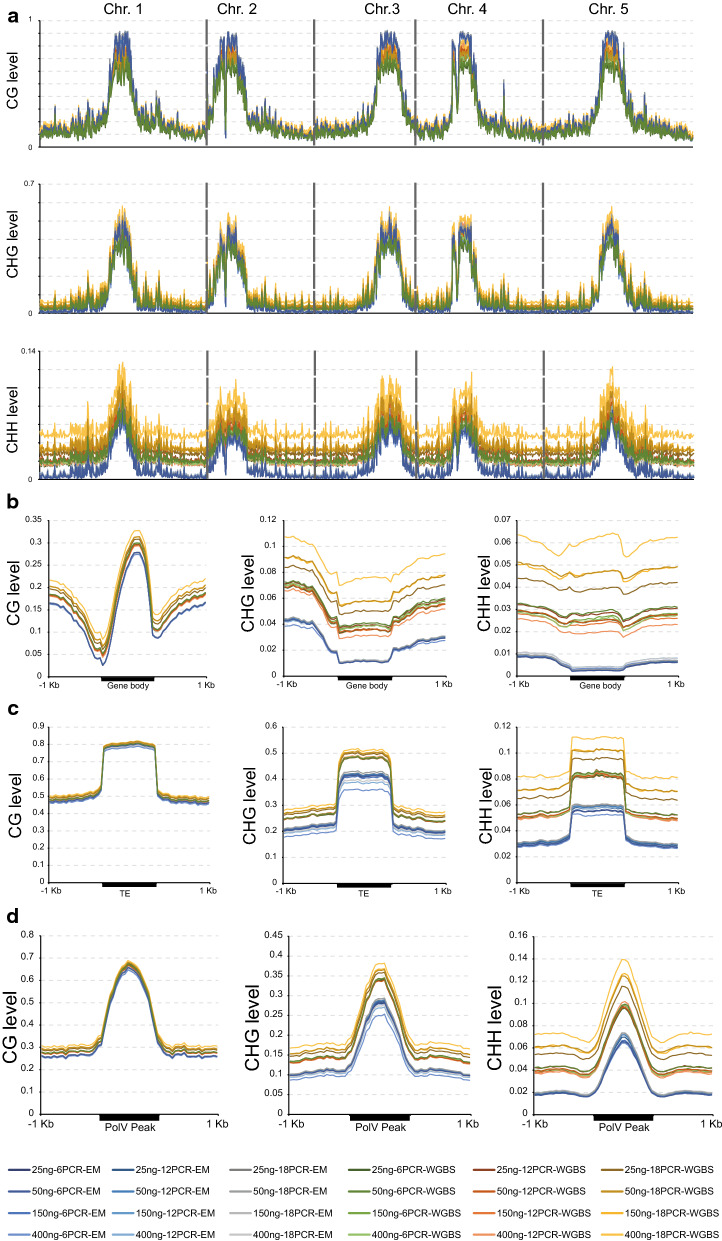
Comparison of methylation pattern between EM-seq and WGBS. **a** Chromosomal distribution of CG, CHG, and CHH methylations. Methylation levels are calculated in Arabidopsis chromosomes divided into 100 Kb bins. **b** Metaplots of CG, CHG, and CHH methylations over genes and 1 Kb flanking sequences. **c** Metaplots of CG, CHG, and CHH methylations over TEs and 1 Kb flanking sequences. **d** Metaplots of CG, CHG, and CHH methylations over PolV ChIP-seq peaks and 1 Kb flanking sequences. In **a**–**d**, colored lines represent different library preparations

We next plotted both EM-seq and WGBS data across all five Arabidopsis chromosomes (Fig. 4a). In general, methylation levels reported by WGBS are higher than those from EM-seq, especially in the case of CHH methylation, where some WGBS libraries made under suboptimal conditions (e.g. higher number of PCR cycles) suffer from severe over-estimation of methylation in the euchromatic arms of the chromosomes. Interestingly, Fig. 4a also reveals that methylation levels measured by EM-seq are higher in pericentromeres than those measured by WGBS. In the next section, we explore this further using differentially methylated region (DMR) analysis.

Arabidopsis has been shown to have two distinctive DNA methylation patterns: CG methylation in the body of protein-coding genes and all three types of methylation (CG, CHG, and CHH) in repetitive DNAs such as transposable elements (TEs) [8, 9, 28–30]. In the past, metaplot analysis by WGBS has always reported some residual non-CG methylation inside gene bodies that were indistinguishable from noise [8, 9] due to the background non-conversion issues associated with bisulfite conversion (Fig. 1c, Additional file 1: Fig. S2). Since we now know that EM-seq has much lower background than WGBS, we ran the same metaplot analysis with EM-seq data and observed much reduced CHG and CHH methylation levels across gene bodies (Fig. 4b). The observed levels were still an order of magnitude higher than the pure background noise that can be inferred from chloroplast methylation, especially in the case of CHG (1.16% CHG methylation on average over gene body compared to 0.22% background) (Additional file 1: Fig. S2, Additional file 2: Table S1c), consistent with the known presence of low levels of non-CG methylation in gene bodies [31]. In terms of methylation in TEs, EM-seq produces similar metaplot profiles as WGBS, albeit with lower levels (especially for CHG and CHH (Fig. 4c), which is expected since WGBS tends to overestimate CHG and CHH methylations (Fig. 3, Additional file 3: Table S2)).

We also compared the methylation patterns and levels in the chromosomal plots and metaplots containing only the best WGBS library (400 ng input with 12 cycles of PCR) and its corresponding EM-seq library (Additional file 1: Fig. S3). As expected, the methylation differences between this pair of libraries were smaller than the differences when other WGBS libraries are included in the comparison. Nevertheless, the basic patterns are the same as described above (see Fig. 4).

Since CHG and CHH methylations are maintained by RNA-directed DNA methylation (RdDM) [29], we looked at methylation in genomic regions bound by POLYMERASE V (PolV), which are often used as a proxy for RdDM target loci [32, 33]. CHG and CHH methylations over the PolV ChIP-seq peaks were elevated to various extents in different WGBS libraries, while all the EM-seq libraries show similar levels (Fig. 4d).

As an example of a gene with a well-studied methylation pattern, we looked at methylation in the *FLOWERING WAGENINGEN* (*FWA*) locus, a target of RdDM [34–36]. While methylated cytosines in non-CG contexts were detected at low levels throughout the *FWA* gene in almost all of the WGBS datasets, EM-seq data clearly show that non-CG methylation only exists in the promoter/beginning of coding sequence (CDS) region of *FWA* (Additional file 1: Fig. S4a), where the known patch of RdDM is known to occur. Even when using only data from the best WGBS library (400 ng input with 12 cycles of PCR), we still see trace amounts of non-CG methylation downstream of the promoter/beginning of CDS region of *FWA*; the same places show no non-CG methylation in EM-seq data (Additional file 1: Fig. S4b).

### Differentially methylated region analyses in EM-seq and WGBS

We performed pairwise differential methylation region (DMR) analysis both within the various datasets of EM-seq or WGBS and across EM-seq and WGBS datasets. Orders of magnitude fewer DMRs are called within the EM-seq libraries than within WGBS libraries (Additional file 1: Fig. S5ab). A larger number of DMRs arose in comparisons between the EM-seq libraries made from the least input DNA amount and the most input DNA amount and between the EM-seq libraries made with the lowest number of PCR cycles and the highest number of PCR cycles (Additional file 1: Fig. S5a). The same trends were observed in comparisons between WGBS libraries, although the WGBS comparisons produce much larger numbers, especially in the case of CHG and CHH methylation (Additional file 1: Fig. S5b).

**Fig. 5:**
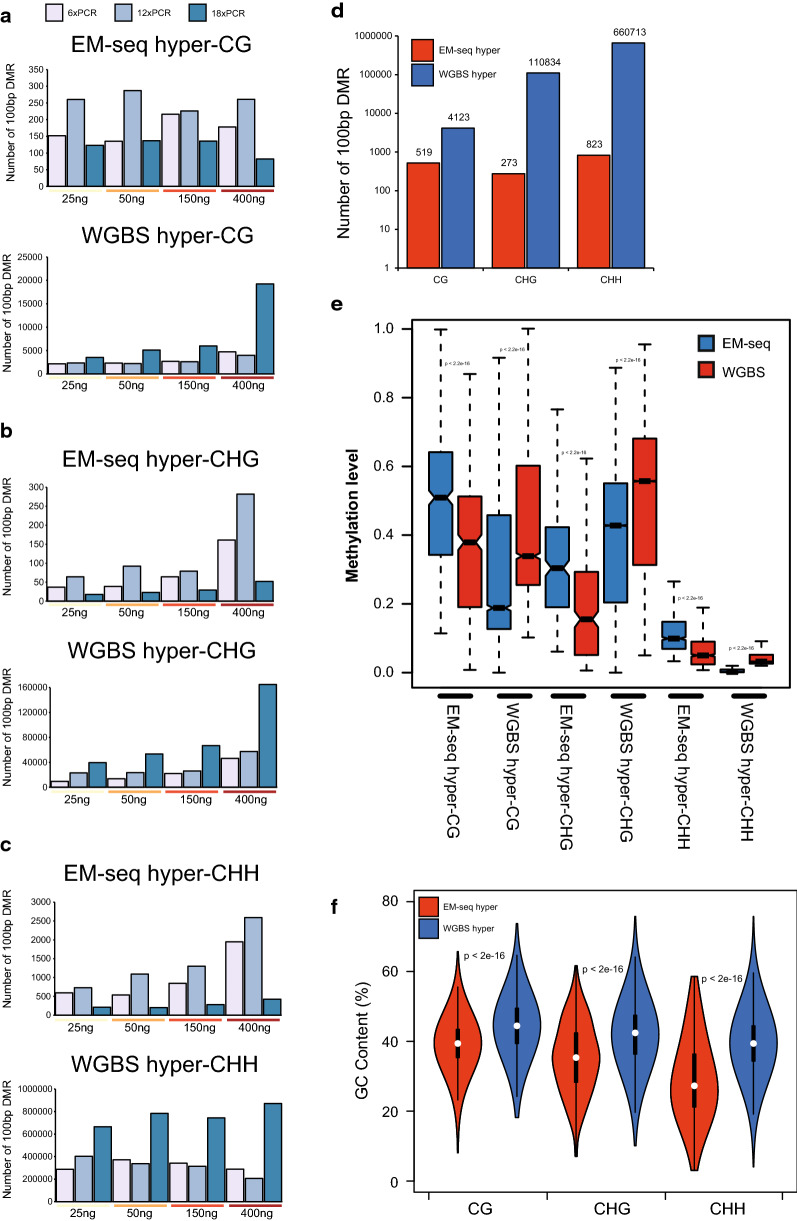
Differences in differentially methylated regions (DMRs) between EM-seq and WGBS. **a**–**c** CG (**a**), CHG (**b**), and CHH (**c**) hyper-DMR numbers of pairwise comparison between WGBS and EM-seq from each of the different library preparation conditions. **d** Total DMR numbers of all EM-seq vs. all WGBS. **e** Methylation levels of EM-seq and WGBS hyper-DMRs. **f** GC contents of EM-seq and WGBS hyper-DMRs. In **e** and **f**, the *P* values, shown at the top of the plots, were estimated using a Student’s *t*-test

When comparing called DMRs between EM-seq libraries and WGBS libraries made from the same amount of input DNA and with the same number of PCR cycles, we noticed that there are many more WGBS hyper-DMRs (higher methylation in WGBS libraries) than EM-seq hyper-DMRs (Fig. 5a–c). WGBS libraries with 18 cycles of PCR were clearly the outliers, since they tended to have more WGBS hyper-DMRs than other conditions, and the situation is made worse by combining 18 cycles of PCR with 400 ng of input DNA (Fig. 5a–c). Therefore, when making WGBS libraries, excess PCR amplification should be avoided, especially if starting with plenty of DNA. There are 7.94 (4123/519) times as many WGBS hyper-CG DMRs as EM-seq hyper-CG DMRs, while the numbers for CHG and CHH are 405.99 (110834/273) and 802.81 (660713/823) times, respectively, suggesting that WGBS has more enrichment of CHG and CHH hyper-DMRs than CG hyper-DMRs (Fig. 5d, Additional file 4: Table S3). This fits with our previous finding that CHG and CHH methylations are more over-estimated by WGBS than is CG methylation (Figs. 3, 4). For EM-seq hyper-DMRs, we saw some variation in DMR numbers among different library preparation conditions, for example, more hyper-DMRs were seen between libraries with 12 cycles of PCR (Fig. 5a–c); however, we suspect that this is rather due to the variation in WGBS libraries than the difference between WGBS and EM-seq libraries (since WGBS libraries have much higher variation among themselves than do EM-seq libraries (Additional file 1: Fig. S5ab)).

We next plotted the methylation levels in the defined EM-seq and WGBS hyper-DMRs (Fig. 5e). Interestingly, EM-seq hyper-CG and CHH DMRs on average have higher methylation levels than WGBS hyper-CG and CHH DMRs, respectively. The methylation level in EM-seq hyper-CHG DMRs is lower than that in WGBS hyper-CHG DMRs, which is the opposite of what is observed in EM-seq hyper-CG and CHH DMRs (Fig. 5e). There are very few EM-seq hyper-CHG DMRs, and many of them are obtained from comparison of EM-seq and WGBS in two conditions (400 ng input, 6 and 12 cycles) (Fig. 5b), which could skew the result. Furthermore, most of the WGBS hyper-CHG DMRs are in pericentromeric heterochromatin regions (Additional file 1: Fig. S5c) with high levels of methylation (see Fig. 4a). GC content analyses in DMRs indicate that EM-seq hyper-DMRs have significantly lower GC content than WGBS hyper-DMRs (Fig. 5f). One extreme case is the mitochondria chromosome (chrM), which has a ~ 10% higher GC content than the five nuclear chromosomes (Additional file 1: Fig. S6a). The relative difference in methylation levels between EM-seq and WGBS in chrM is larger than that in other chromosomes (Additional file 1: Fig. S6b), and in fact the majority of the chrM is called as WBGS hyper-DMRs (Additional file 5: Table S4). The methylation levels in chrM are generally very low as determined by EM-seq (Additional file 1: Fig. S6b), which fits with the previous observation that WGBS hyper-DMRs tend to have lower methylation levels (Fig. 5e). We reasoned that in WGBS hyper-DMRs in nuclear chromosomes there are likely many sites that should have no or very low methylation (like chrM); this would make the average methylation levels in WGBS hyper-DMRs low, except for WGBS hyper-CHG DMRs for above-mentioned reasons (see Fig. 5e, Additional file 1: Fig. S5c).

Since GC content also greatly affects coverage (Fig. 2c), we wondered if over- (mainly CHG and CHH) and under-estimating (mainly CG) methylation by WGBS could be linked to coverage. First, we generated heatmaps and coverage plots of all the libraries across PolV ChIP-seq peaks, because these are the places with large increases in CHG and CHH methylation in WGBS libraries (Fig. 4d). We found that for EM-seq libraries, although coverage fluctuates, the ranges are typically quite small. On the other hand, there is a significant increase in coverage coinciding with the center of PolV ChIP-seq peaks for all WGBS libraries (Additional file 1: Fig. S7). A reasonable explanation for this is that PolV-binding sites are targets of RdDM and contain methylated CHGs and CHHs that are not converted by bisulfite treatment; they, therefore, become better templates for PCR amplification (see data from previous sections) and gain higher coverage than their surrounding regions. Therefore, methylation levels and coverage are positively correlated in this case. The majority of the EM-seq hyper-CG DMRs are located within pericentromere heterochromatins and are highly methylated (Fig. 4a, Fig. 5e, Additional file 1: Fig S8a), but occasionally they can be found in euchromatin regions and within genes (Additional file 1: Fig. S8b). We chose the best WGBS library (400 ng input with 12 cycles of PCR) and its corresponding EM-seq library and analyzed their coverage across EM-seq hyper-DMRs (Additional file 1: Fig. S8c–e). Interestingly, WGBS coverage spikes in EM-seq hyper-DMRs as well, and EM-seq coverage again shows only small fluctuations. The low GC content of EM-seq DMRs (Fig. 5f) could be the cause of high coverage in WGBS libraries (see Fig. 2c). Moreover, according to Fig. 2c, in regions where GC content is low (~ 30% or less, approximately the range of GC content found in EM-seq hyper-DMRS, see Fig. 5f), a further reduction in GC content (e.g., that caused by bisulfite treatment) will induce a sharp increase in WGBS coverage. This effect likely outweighs the PCR preference for unconverted DNA and causes WGBS to under-estimate methylation levels in these regions.

### Methylation differences between Arabidopsis leaves and flowers detected by EM-seq

**Fig. 6:**
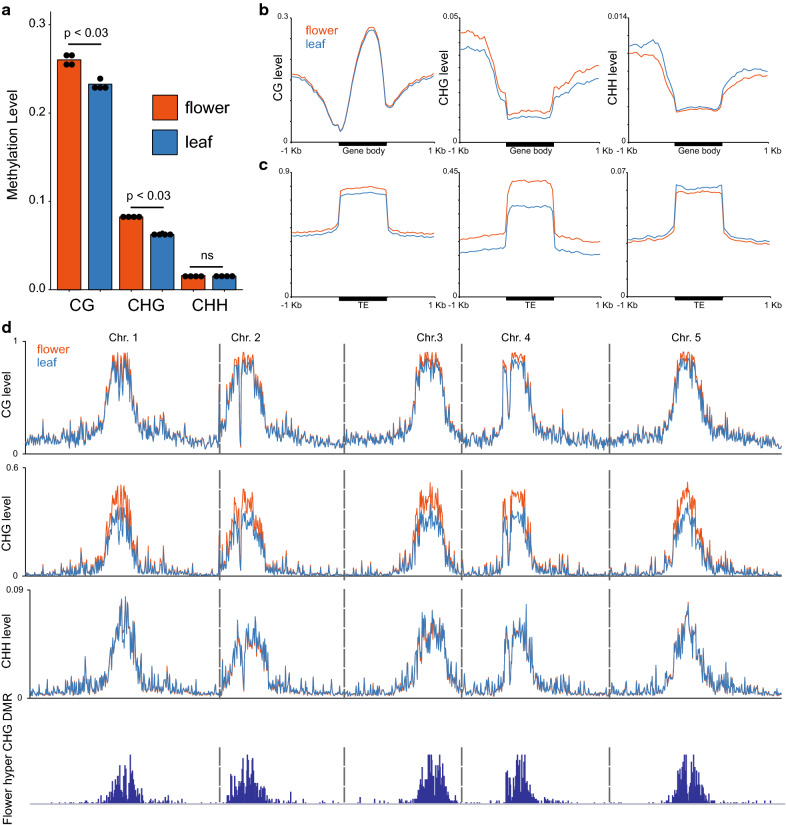
Methylation levels in Arabidopsis leaf and flower samples detected by EM-seq. **a** Whole-genome CG, CHG, and CHH methylation levels of Arabidopsis leaf and flower samples. **b** Metaplots of CG, CHG, and CHH methylations in leaf and flower samples over genes and 1 Kb flanking sequences. **c** Metaplots of CG, CHG, and CHH methylations in leaf and flower samples over TEs and 1 Kb flanking sequences. **d** Chromosomal distribution of CG, CHG, and CHH methylations and CHG DMRs in leaf and flower samples. Methylation levels are calculated in Arabidopsis chromosomes divided into 100 Kb bins

**Fig. 7:**
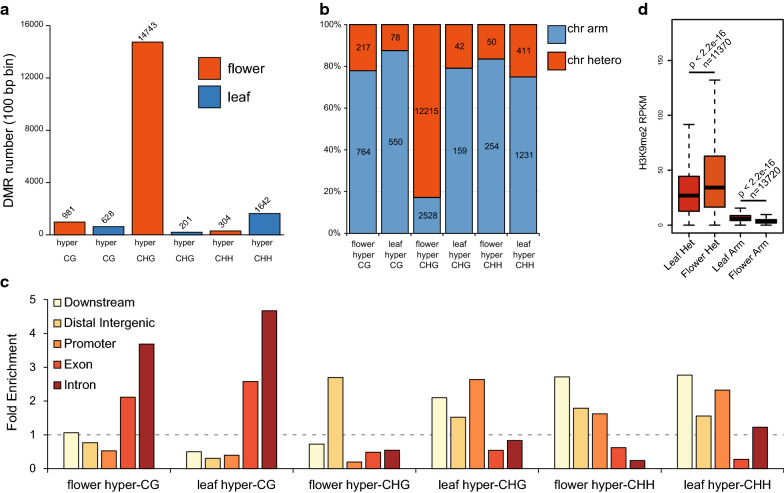
DMRs and H3K9me2 levels in leaf and flower samples. **a** Total number of CG, CHG, and CHH hyper-DMRs in flower and leaf samples. **b** Distribution of CG, CHG, and CHH hyper-DMRs in chromosomal arms and heterochromatins. **c** Enrichment of CG, CHG, and CHH hyper-DMRs in various regions relative to genes. Expected level (1, marked by dotted line) is estimated based on genomic distribution of randomly selected regions of equal size from Arabidopsis genome. **d** H3K9me2 levels in TEs from chromosomal arms (without flower hyper-CHG DMRs) and heterochromatins (with flower hyper-CHG DMRs)

We applied the EM-seq method to analyze methylation differences between Arabidopsis leaves and flowers. We used 150 ng input DNA and 6 cycles of PCR, and generated 4 replicates for each tissue. Overall, CG and CHG methylation levels were slightly higher in flowers than in leaves, and CHH methylation was about the same in both tissues (Fig. 6a). Metaplots in genes reveal that there are very small differences between leaves and flowers in gene body methylation levels–CG plots are almost identical and CHG and CHH are close to noise levels in both tissues (Fig. 6b). For TEs, CG is very slightly increased and CHH is very slightly decreased in flowers compared to leaves (Fig. 6c), and the same trends can be observed in pericentromeric heterochromatins in chromosomal methylation plots (Fig. 6d). The most striking finding from these analyses is that CHG methylation is significantly higher in flowers than in leaves (Fig. 6 cd). Consistent with this, a much larger number of flower hyper-DMRs are called in the CHG context than in CG or CHH (Fig. 7a, Additional file 6: Table S5). Flower hyper-CHG DMRs are located mainly in pericentromeric heterochromatins and not inside or close to genes (Figs. 6d,  7bc). DMRs for non-CG methylations are not enriched within genes, as non-CG methylations are usually not found in the genes (Figs. 6b, 7c).

CHG methylation is mainly mediated by CHROMOMETHYLASE2 (CMT2) and CMT3 via a self-reinforcing loop involving histone H3K9 methylation [22, 37, 38]. Indeed, when examining the transposons in heterochromatin regions (that have flower hyper-CHG DMRs), we observed a higher level of H3K9me2 by ChIP-seq in flowers than in leaves, whereas there was very little change in the H3K9me2 levels of euchromatic TEs that did not show flower hyper-CHG DMRs (Fig. 7d). We note that the absolute level of H3K9me2 is much higher in heterochromatin regions than in chromosome arms (Fig. 7d), which is a characteristic of the Arabidopsis epigenome [39]. These results are consistent with a more active CHG and H3K9me2 self-reinforcing loop in flowers affecting heterochromatic TEs.

Although the overall differences in CG methylation within gene bodies are minimal between leaves and flowers (Fig. 6b), hyper-CG DMRs from both flowers and leaves were enriched in gene exons and introns (Fig. 7c; see an example in Additional file 1: Fig. S9a). Indeed, when we plotted CG methylation on genes with hyper-CG DMRs in either leaves (Additional file 1: Fig. S9b, left panel) or flowers (Additional file 1: Fig. S9b, right panel), we observed clear differences. Previous studies have shown that gene body-methylated genes in plants are often house-keeping genes, constitutively expressed, and exhibit moderately high expression levels [28, 40, 41]. We found that majority of the genes with hyper-CG methylations in either leaves or flowers are differentially expressed between leaves and flowers (Additional file 1: Fig. S9c). However, this does not seem to be specific, as we obtained similar results when looking at randomly selected genes (data not shown). This and the fact that both upregulated and downregulated genes in both tissues can show increased body CG methylation (Additional file 1: Fig. S9c) suggests that gene body methylation does not directly regulate the expression of these genes.

## Discussion

Bisulfite sequencing, despite being the gold standard for methylation detection, is known to have shortcomings, including DNA damage, false positives due to non-conversion, uneven and missing coverage, and biased representation of methylated versus unmethylated DNA in the final library. In this study, we performed detailed analyses of these aspects and compared the results from WGBS to those from EM-seq, a newly developed, enzyme-based, bisulfite-free method for methylation detection. Our WGBS findings are consistent with those in previously published literature [7, 11–13, 21, 25–27]. In all the comparisons between EM-seq and WGBS, EM-seq appears to be mostly free of the problems that WGBS has, suggesting that it is a superior method. In addition, library preparation conditions such as input DNA amount and number of PCR amplification cycles have very little effect on the results of EM-seq, while the results of WGBS are greatly affected by these parameters, even when all WGBS libraries are generated in a single batch. Therefore, we propose that EM-seq is more desirable than WGBS, especially for big data projects that require integration of datasets obtained across a wide variety of source materials, locations and time points, and processed by personnel with different levels of expertise.

Because of its high reproducibility and low background, EM-seq is also suitable for projects aimed at revealing subtle methylation changes in different samples and/or conditions. We used EM-seq to study methylation differences between Arabidopsis leaves and flowers, and showed that DMRs can be called with high confidence even in places where the two tissues have very similar methylation levels. This approach can be expanded to other tissues and across organisms. EM-seq can work with much lower amounts of starting DNA than WGBS (as low as 100 pg [20]), which makes it ideal for single-cell methylome studies. Currently, bisulfite is used in these studies [42, 43]. Based on the performance of EM-seq observed here, we expect that substituting bisulfite with TET2 and APOBEC3A will greatly enhance the success rate of single-cell methylome library generation and increase the coverage per cell.

It is worth noting that after methylated cytosines are oxidized by TET family enzymes to 5-fCs and 5-caCs, a different approach can be taken to differentiate methylated from unmethylated cytosines. In a recently published TET-assisted pyridine borane sequencing (TAPS) procedure [44], TET1 is used in the first step (to catalyze a similar reaction to that catalyzed by TET2 in EM-seq), and pyridine borane is used in the second step to convert 5-fCs and 5-caCs to dihydrouracils (DHU). DHUs basepair with adenines during PCR and, thus, are amplified as thymines. A set of new bioinformatics tools is needed for TAPS, since in TAPS data methylated cytosines appear as thymines and unmethylated cytosines stay as cytosines, which is the opposite to WGBS and EM-seq. TAPS shows promise in overcoming many of the issues of WGBS; however, since it introduces a different skewed base composition landscape for the current library preparation reagents, sequencers, and bioinformatics tools to deal with, it could potentially lead to complications.

There are new developments in long-read sequencing technologies that enable direct sequencing of the original DNA without fragmentation or amplification, thus bypassing the need for bisulfite treatment. 5-methylcytosines can be differentiated from other bases by the virtue of distinct polymerase kinetics [45, 46] or unique electronic signal characteristics [47, 48] displayed by different bases. Nevertheless, WGBS still compares favorably in terms of accuracy, reliability, and cost effectiveness, against these technologies, at least in their current iterations [49, 50]. An interesting alternative to improve upon these technologies is to combine bisulfite or enzymatic-based conversion with these long-read technologies. For example, bisulfite treatment has been used in combination with PacBio SMRT sequencing [51] and TAPS has been used with both SMRT and Oxford Nanopore sequencing [50]. TET2 and APOPEC3A from the EM-seq protocol can likely be adapted to the same long-read technologies, and because of their non-destructive nature, they are expected to better preserve the intactness of high-molecular weight genomic DNA. However, in both of the previously published methods, because sequencing of PCR products containing newly formed thymines (which correspond to unmethylated cytosines following bisulfite treatment or methylated cytosines following TAPS treatment) outperforms sequencing of intermediates (like oxidized methylcytosines, uracils, or DHUs) in terms of sensitivity, accuracy, and minimal coverage required, the converted DNAs have to be amplified with region-specific primers (aiming to obtain products up to 10 kilobases long) before being subjected to SMRT or Nanopore sequencing. This makes it impractical to use these methods for whole-genome methylation measurement of most eukaryotic organisms. Further advancements in long-read sequencing technologies to allow unequivocal identification of the products of bisulfite, TETs, pyridine borane, or APOBECs are needed for true high efficiency, amplification-free global detection of methylation.

## Conclusion

Enzymatic methyl-seq (EM-seq) uses non-destructive enzymatic reactions, utilizing TET2 and APOBEC3A to convert unmethylated (but not methylated) cytosines to uracils. This approach generates the same product as bisulfite treatment, which can then be sequenced and analyzed in the same way. Here, we showed that compared to whole-genome bisulfite sequencing (WBGS), EM-seq has a higher mapping rate, lower duplication rate, and lower false-positive rate. EM-seq not only displays higher coverage than WGBS, but also the coverage is less affected by GC content. In terms of methylation detection, EM-seq covers more cytosines than WGBS and does not over-estimate methylation levels as WGBS does, especially in the context of CHG and CHH. EM-seq exhibits better consistency within libraries made from the same materials in all quality aspects examined and in report of methylation levels. Thus, in many respects, EM-seq is superior to WGBS.

## Methods

### Plant materials

Arabidopsis plants of the Columbia-0 (Col-0) ecotype were used in this study. All plants were grown at 22 °C in a long day (16-h light, 8-h dark) growth room. Flowers and leaves were collected from 4- to 5-week-old plants.

### Genomic DNA extraction and fragmentation

Genomic DNA from Arabidopsis flowers and leaves was extracted using a DNeasy Plant Mini Kit (Qiagen). Concentration of the DNA was measured by Qubit dsDNA Broad-Range Assay kit (ThermoFisher). 50 μl aliquots containing 25, 50, 150, and 400 ng DNA were sheared by an S2 Focused-ultrasonicator (Covaris) to ~ 200 bp in average size using these parameters: intensity 5, duty cycle 10%, cycles per burst 200, treatment time 120 s.

### EM-seq library preparation

EM-seq libraries were prepared from sheared DNA using an enzymatic methyl-seq kit following the manufacturer instructions (New England BioLabs). For each input amount, three PCR conditions were used: 6, 12, and 18 cycles.

### Whole-genome bisulfite library (WGBS) preparation

Sheared DNA was end-repaired and ligated with TruSeq DNA single adapters (Illumina) using a Kapa DNA HyperPrep kit (Roche). Adapter-ligated DNA was converted with an EpiTect Bisulfite Kit (Qiagen). Converted DNA was PCR-amplified by MyTaq polymerase (Bioline) for 6, 12, or 18 cycles.

### Processing and sequencing of EM-seq and WGBS libraries

The libraries were run on D1000 ScreenTape (Agilent) to determine the quality and size. The libraries were then purified by AMPure XP beads (Beckman Coulter) and concentrations were measured with a Qubit dsDNA Broad-Range Assay kit (ThermoFisher). Finally, libraries were sequenced on a NovaSeq 6000 sequencer (Illumina) to obtain single-end 100 bp reads.

### Single-nucleotide resolution methylome mapping

WGBS and EM-seq reads were trimmed with trim_galore (v0.4.2) (https://www.bioinformatics.babraham.ac.uk/projects/trim_galore/). Adapter trimmed reads were mapped to TAIR10 reference genome by BSMAP (v2.90) allowing 2 mismatches and 1 best hit (-v 2 -w 1) [52]. Reads with three or more consecutive methylated CHH sites were considered as unconverted reads and subsequently removed in the following analysis. Mapping and duplication rates were obtained from log files of the BSMAP pipeline.

### Single-nucleotide resolution methylome level calculation

DNA methylation level at each site or region was calculated by number of methylated C vs. total C and T account. False-positive methylation levels were estimated by calculating methylation level in the Arabidopsis chloroplast genome since it is virtually unmethylated. To calculate the methylation level of genes and transposable elements, gene body or transposable element regions were divided into 20 proportionally sized bins and up/down-stream 1 kb regions into 50 bp bins. Genome-wide average methylation level of genes and transposon elements was then calculated at these bins.

### Single-nucleotide resolution methylome DMR calling and annotation

Differentially Methylated Regions (DMRs) were called by methdiff.py in BSMAP with *P* < 0.01 where differences in CG, CHG, and CHH methylation were at least 0.1, 0.05, and 0.02 (WGBS and EM-seq comparison) or 0.4, 0.2, and 0.1 (flower and leaf EM-seq comparison), respectively. Genomic distributions of DMRs were annotated by ChIPseeker [53]. Control datasets were obtained by randomly selected, equal length regions in TAIR10 genome with the bedtools shuffle function (v2.27.1). Gene expression profile was downloaded from the TAIR database (https://www.arabidopsis.org/) and visualized with R package pheatmap [54]. Chromosome arm and heterochromatin regions were defined with H3K9me2 ChIP-seq data [37]. Regions highly enriched with H3K9me2 ChIP-seq signal were defined as heterochromatin regions.

### Reads coverage analysis

Reads coverage depth were estimated through converting mapping depth with deeptools2 (v2.5.1) [55] and plotted over PolV ChIP-seq peaks [33] or DMRs. Dinucleotide coverage was calculated with bam2nuc module integrated in Bismark (v0.18.2) [56]. Normalized read coverage related to GC content was estimated by CollectGcBiasMetrics in Picard (v2.13.2) (http://broadinstitute.github.io/picard/). mC density was calculated by dividing the genome into 400 bp bins and counting methylated Cs in each bin in the CG, CHG, or CHH context.

### Chromatin immunoprecipitation (ChIP) assays

H3K9me2 ChIP-seq in Arabidopsis leaves was previously published [57]. For H3K9me2 ChIP-seq in Arabidopsis flowers, 3 grams of Arabidopsis Col-0 wild-type unopened flower buds were collected. The nuclei were isolated from these materials for in vitro cross-linking with 1% formaldehyde. Nuclei were lysed and the chromatin was sheared with Bioruptor Plus (Diagenode). The sheared chromatins were equally separated for two ChIPs. 5 μl of anti-H3K9me2 (ab1220, abcam) and anti-H3 (ab1791, abcam) antibodies were added for chromatin immunoprecipitation, respectively. This experiment was performed by closely following the protocol described in a previous paper [58]. ChIP-seq libraries were prepared from DNA extracted from the ChIP experiment using Ovation Ultra Low System V2 Kit following manufacturer instructions (NuGEN). The libraries were sequenced on a HiSeq 4000 sequencer (Illumina) to obtain single-end 50 bp reads. To assess differences in H3K9me2 level at heterochromatin and euchromatin regions, we selected heterochromatin TEs that overlap with flower hyper-CHG DMRs and euchromatic TEs that do not overlap with flower hyper-CHG DMRs and compared their H3K9me2 level in leaf and flower tissue, respectively. H3K9me2 levels were calculated by converting ChIP seq reads count to RPKM with bamCoverage function in bedtools [59].

## Supplementary information

**Additional file 1: Figure S1.** Schematic diagram of experimental design. **Figure S2** CG, CHG, and CHH methylation levels detected in chloroplast genome by EM-seq and WGBS. Blue box represents EM-seq and red box represents WGBS. The P values, shown at the top of the plots, were estimated with a Student’s *t* test. **Figure S3** Comparison of methylation pattern between EM-seq and WGBS libraries prepared with 400 ng DNA input and 12 cycles of PCR. **a** Chromosomal distribution of CG, CHG, and CHH methylations. Methylation levels were calculated with Arabidopsis chromosomes divided into 100 Kb bins. **b** Metaplots of CG, CHG, and CHH methylations over genes and 1 Kb flanking sequences. **c** Metaplots of CG, CHG, and CHH methylation over TEs and 1 Kb flanking sequences. **Figure S4** Genome browser screenshots of *FWA* locus. **a** Comparison of methylation detected by EM-Seq and WGBS in libraries prepared with 50 ng DNA input. **b** Comparison of methylation detected by EM-Seq and WGBS in libraries prepared with 400 ng DNA input and 12 cycles of PCR. **Figure S5** DMR numbers and chromosomal distribution. **a** DMR numbers between EM-seq libraries prepared with different conditions. **b** DMR numbers between WGBS libraries prepared with different conditions. **c** Chromosomal distribution of CHG methylation in EM-seq and WBGS libraries prepared with 400 ng DNA input and 12 cycles of PCR and WGBS hyper-CHG DMRs. **Figure S6** Methylation in mitochondrial DNA. **a** GC contents of Arabidopsis chromosomes. **b** CG, CHG, and CHH methylation levels in mitochondrial DNA. **Figure S7** Heatmaps showing read coverage of EM-seq (left six columns both rows) and WGBS (right six columns both rows) over PolV ChIP-seq peaks.**Figure S8** EM-seq hyper-DMRs. **a**,**b** Genome browser screenshots of EM-seq hyper-DMRs in pericentromeric TE (**a**) and in gene (**b**). **c**–**e** Heatmaps showing read coverage of EM-seq (left panels) and WGBS (right panels) over EM-seq hyper-CG (**c**), CHG (**d**), and CHH (**e**) DMRs. **Figure S9** Gene body methylated genes. **a** Genome browser screenshots of flower hyper-CG DMRs in a gene body methylated gene. **b** Metaplots of CG, CHG, and CHH methylations in leaf and flower samples over genes containing hyper-CG DMRs and 1 Kb flanking sequences. **c** Clustering of expression patterns of gene body methylated genes containing hyper-CG DMRs.

**Additional file 2: Table S1.** Comparison of sequencing results from EM-seq and WGBS libraries. **a** Mapping rates and duplication rates. **b** Non-conversion filtering rates and coverages. **c** Methylation levels in chloroplast DNA.

**Additional file 3: Table S2.** Whole-genome methylation levels in EM-seq and WGBS.

**Additional file 4: Table S3.** DMRs between EM-seq and WGBS

**Additional file 5: Table S4.** DMRs in mitochondrial DNA.

**Additional file 6: Table S5.** DMRs between Arabidopsis leaf and flower samples.

## Data Availability

High-throughput sequencing data generated in this study can be accessed through Gene Expression Omnibus (GEO) database under accession number GSE151616.
