# Supplementary material for: Efficient and accurate determination of genome-wide DNA methylation patterns in Arabidopsis thaliana with enzymatic methyl sequencing
Source: Epigenetics Chromatin. 2020 Oct 7;13:42. doi: 10.1186/s13072-020-00361-9 (PMC7542392; doi:10.1186/s13072-020-00361-9)
Supplement: Supplementary file 1 — Additional file 1: Figure S1. Schematic diagram of experimental design. Figure S2 CG, CHG, and CHH methylation levels detected in chloroplast genome by EM-seq and WGBS. Blue box represents EM-seq and red box represents WGBS. The P values, shown at the top of the plots, were estimated with a Student’s t test. Figure S3 Comparison of methylation pattern between EM-seq and WGBS libraries prepared with 400 ng DNA input and 12 cycles of PCR. a Chromosomal distribution of CG, CHG, and CHH methylations. Methylation levels were calculated with Arabidopsis chromosomes divided into 100 Kb bins. b Metaplots of CG, CHG, and CHH methylations over genes and 1 Kb flanking sequences. c Metaplots of CG, CHG, and CHH methylation over TEs and 1 Kb flanking sequences. Figure S4 Genome browser screenshots of FWA locus. a Comparison of methylation detected by EM-Seq and WGBS in libraries prepared with 50 ng DNA input. b Comparison of methylation detected by EM-Seq and WGBS in libraries prepared with 400 ng DNA input and 12 cycles of PCR. Figure S5 DMR numbers and chromosomal distribution. a DMR numbers between EM-seq libraries prepared with different conditions. b DMR numbers between WGBS libraries prepared with different conditions. c Chromosomal distribution of CHG methylation in EM-seq and WBGS libraries prepared with 400 ng DNA input and 12 cycles of PCR and WGBS hyper-CHG DMRs. Figure S6 Methylation in mitochondrial DNA. a GC contents of Arabidopsis chromosomes. b CG, CHG, and CHH methylation levels in mitochondrial DNA. Figure S7 Heatmaps showing read coverage of EM-seq (left six columns both rows) and WGBS (right six columns both rows) over PolV ChIP-seq peaks.Figure S8 EM-seq hyper-DMRs. a,b Genome browser screenshots of EM-seq hyper-DMRs in pericentromeric TE (a) and in gene (b). c–e Heatmaps showing read coverage of EM-seq (left panels) and WGBS (right panels) over EM-seq hyper-CG (c), CHG (d), and CHH (e) DMRs. Figure S9 Gene body methylated genes. a Genome browser screenshots [file 13072_2020_361_MOESM1_ESM.pdf]

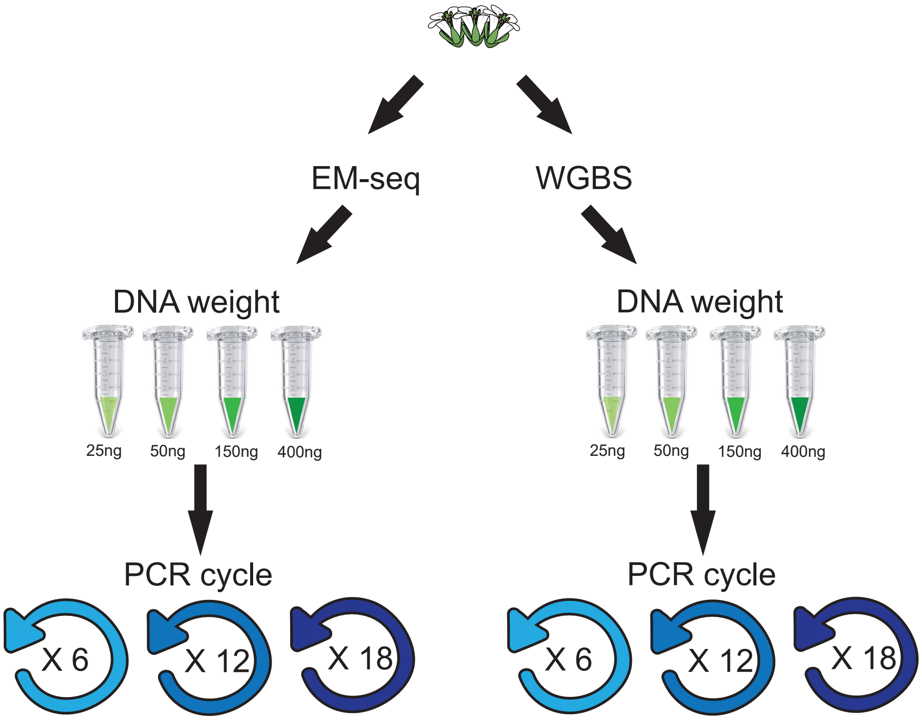

Figure S1 Schematic diagram of experimental design.

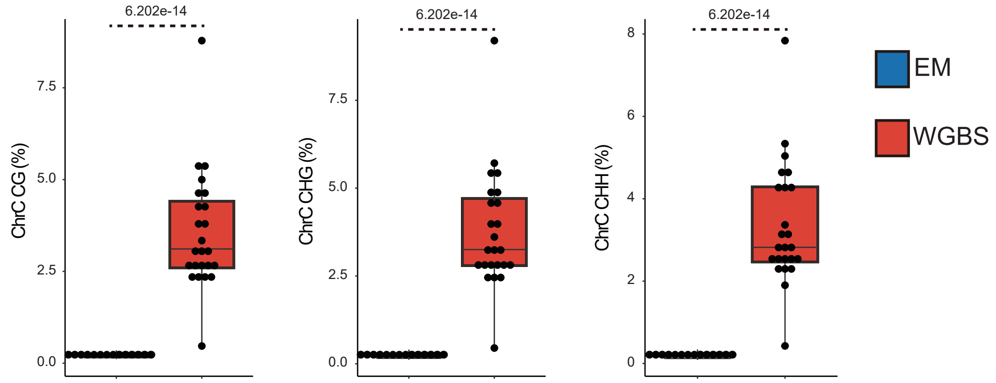

Figure S2 CG, CHG, and CHH methylation levels detected in chloroplast genome by EM-seq and WGBS. Blue box represents EM-seq and red box represents WGBS. The P values, shown at the top of the plots, were estimated with a Student's t-test.

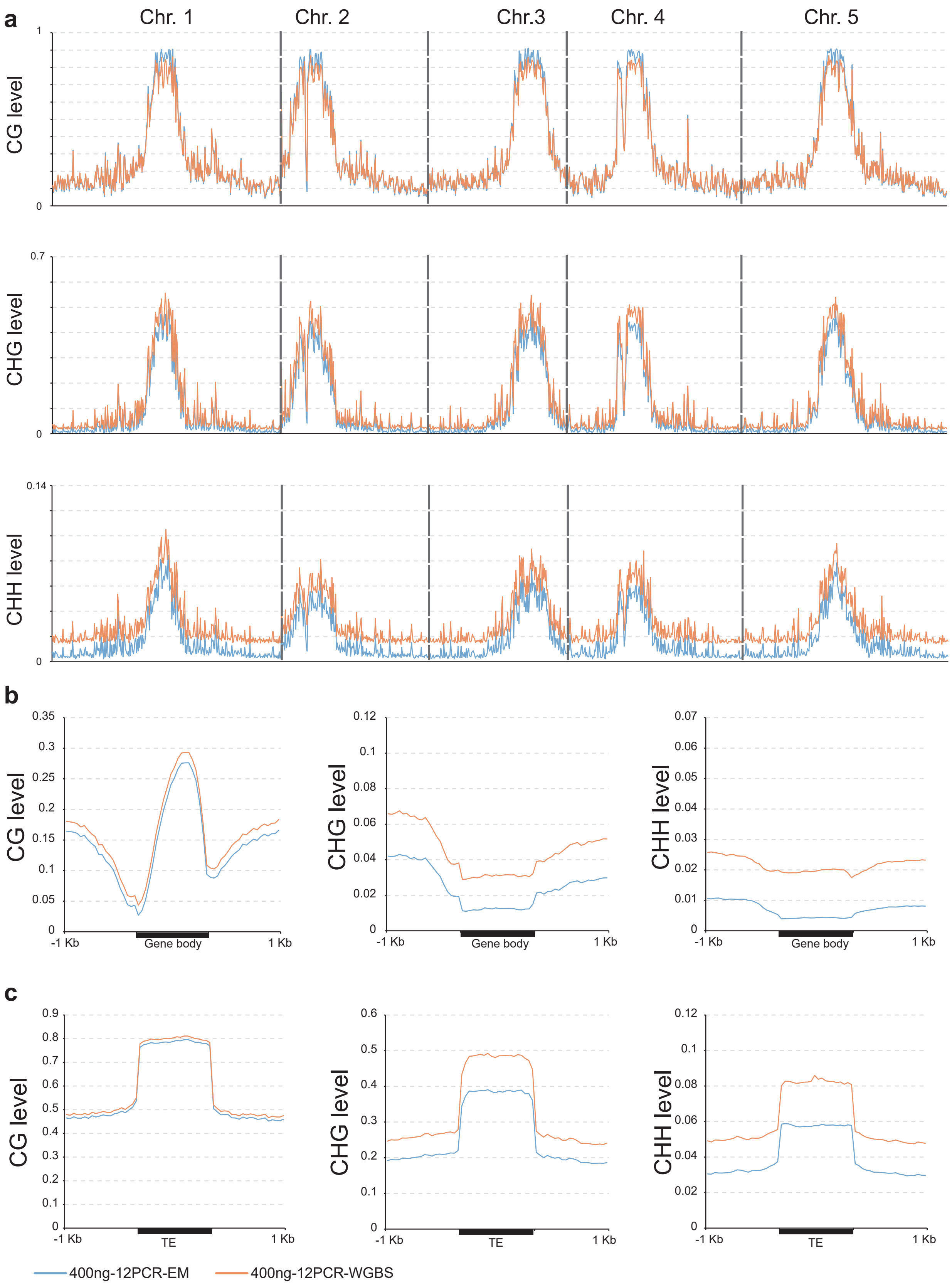

Figure S3 Comparison of methylation pattern between EM-seq and WGBS libraries prepared with 400ng DNA input and 12 cycles of PCR. **a** Chromosomal distribution of CG, CHG, and CHH methylations. Methylation levels were calculated with Arabidopsis chromosomes divided into 100 Kb bins. **b** Metaplots of CG, CHG, and CHH methylations over genes and 1 Kb flanking sequences. **c** Metaplots of CG, CHG, and CHH methylation over TEs and 1 Kb flanking sequences.

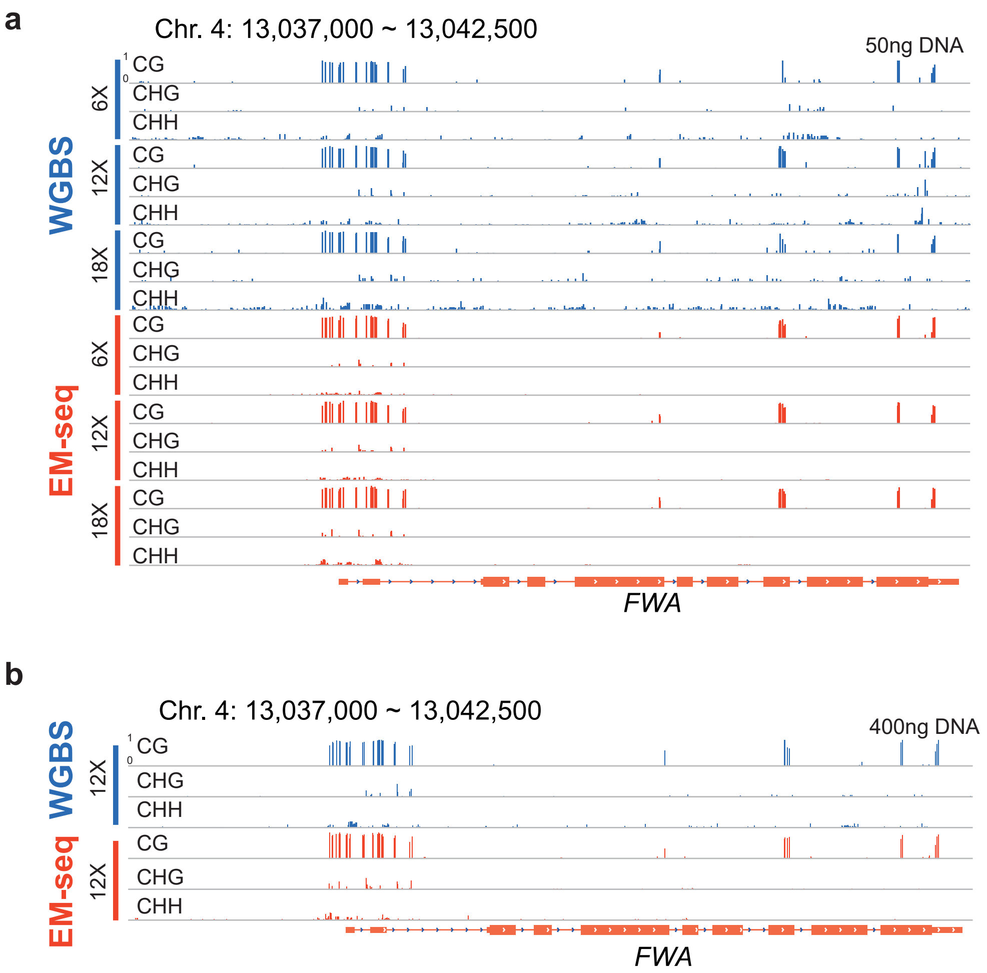

Figure S4 Genome browser screenshots of FWA locus. **a** Comparison of methylation detected by EM-Seq and WGBS in libraries prepared with 50ng DNA input. **b** Comparison of methylation detected by EM-Seq and WGBS in libraries prepared with 400ng DNA input and 12 cycles of PCR.

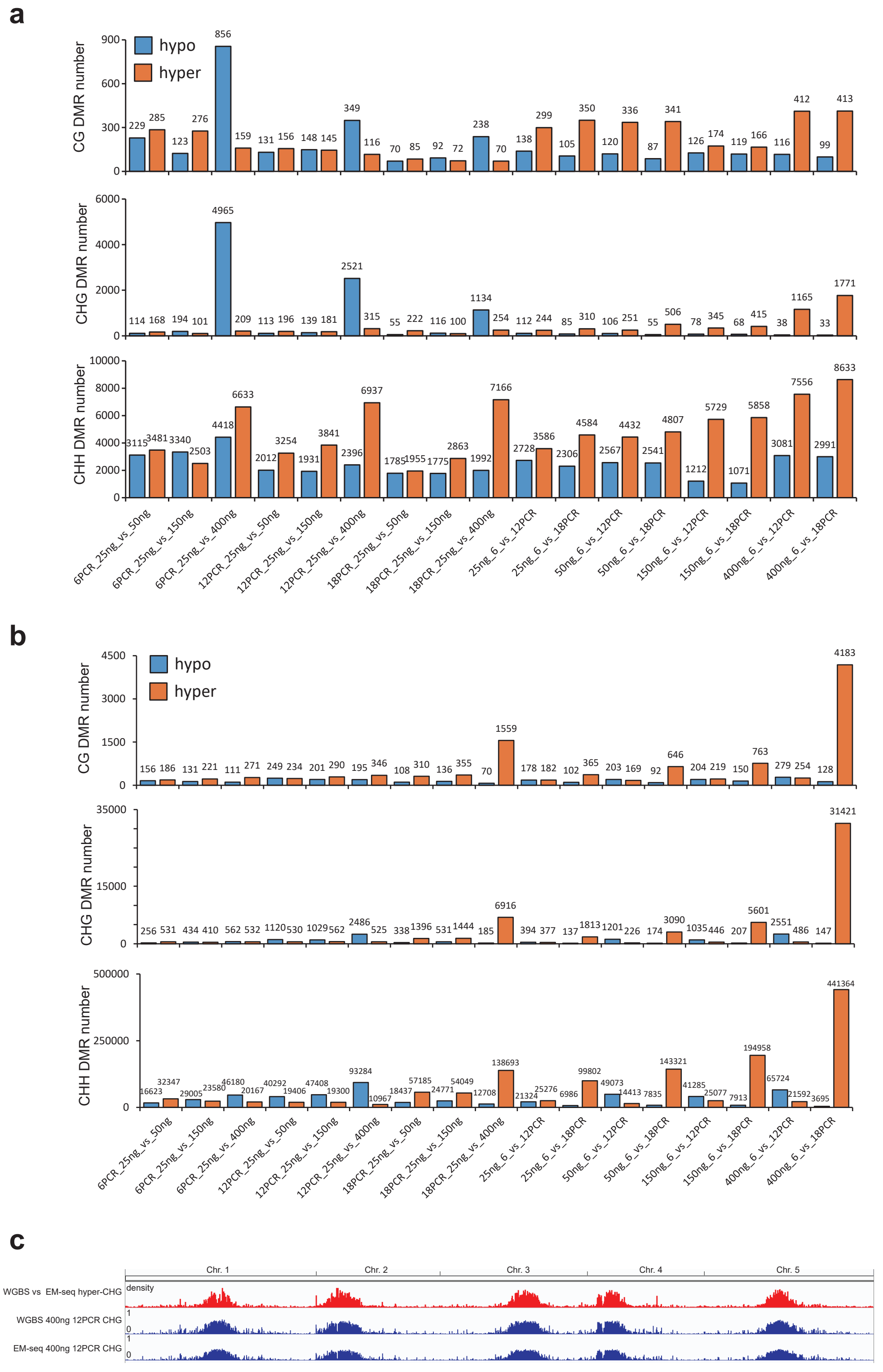

Figure S5 DMR numbers and chromosomal distribution. **a** DMR numbers between EM-seq libraries prepared with different conditions. **b** DMR numbers between WGBS libraries prepared with different conditions. **c** Chromosomal distribution of CHG methylation in EM-seq and WBGS libraries prepared with 400ng DNA input and 12 cycles of PCR and WGBS hyper-CHG DMRs.

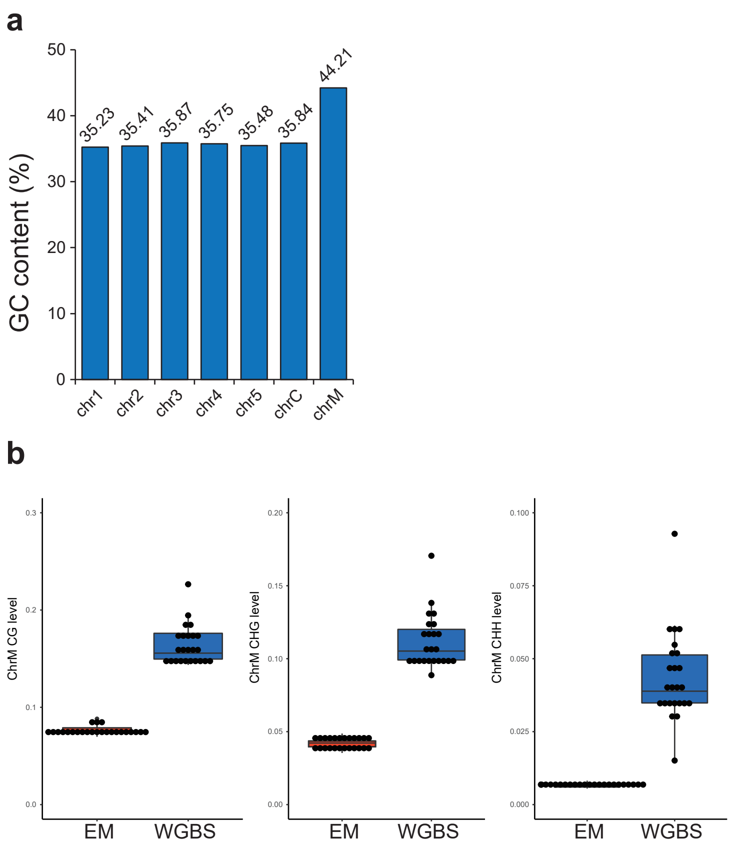

Figure S6 Methylation in mitochondrial DNA. **a** GC contents of Arabidopsis chromosomes. **b** CG, CHG, and CHH methylation levels in mitochondrial DNA.

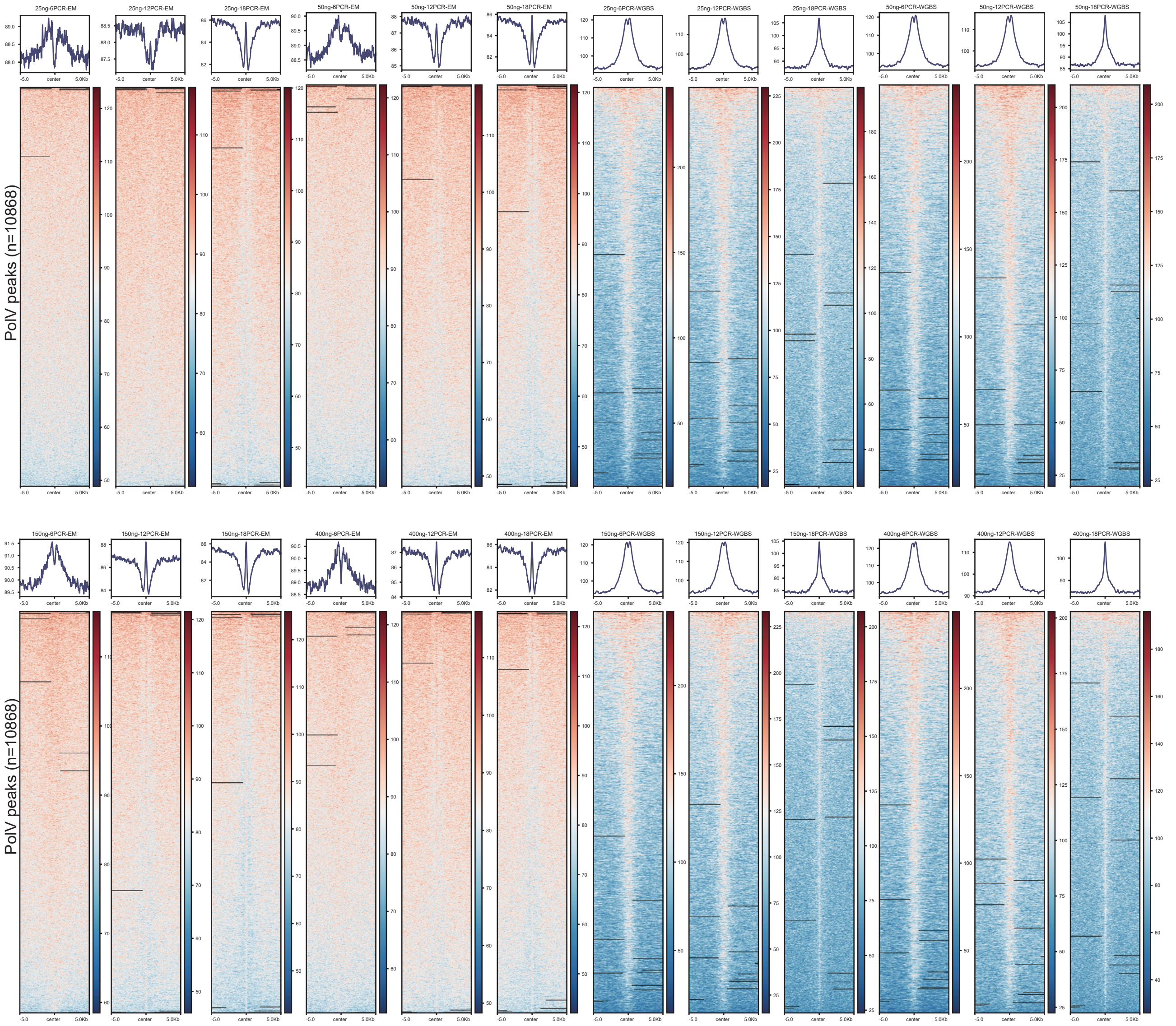

Figure S7 Heatmaps showing read coverage of EM-seq (left six columns both rows) and WGBS (right six columns both rows) over PolV ChIP-seq peaks.

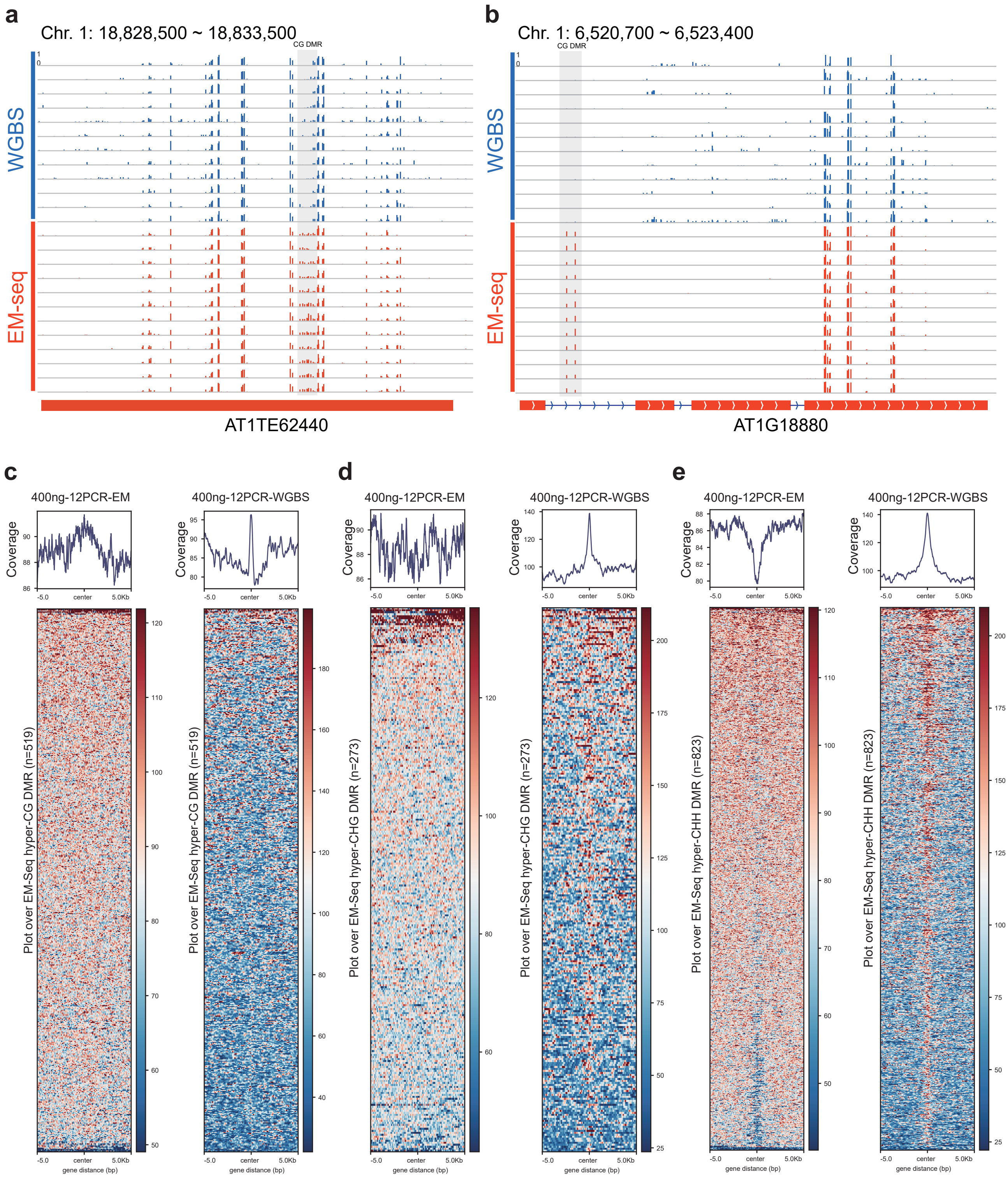

Figure S8 EM-seq hyper-DMRs. **a**,**b** Genome browser screenshots of EM-seq hyper-DMRs in pericentromeric TE (**a**) and in gene (**b**). **c-e** Heatmaps showing read coverage of EM-seq (left panels) and WGBS (right panels) over EM-seq hyper-CG (**c**), CHG (**d**), and CHH (**e**) DMRs.

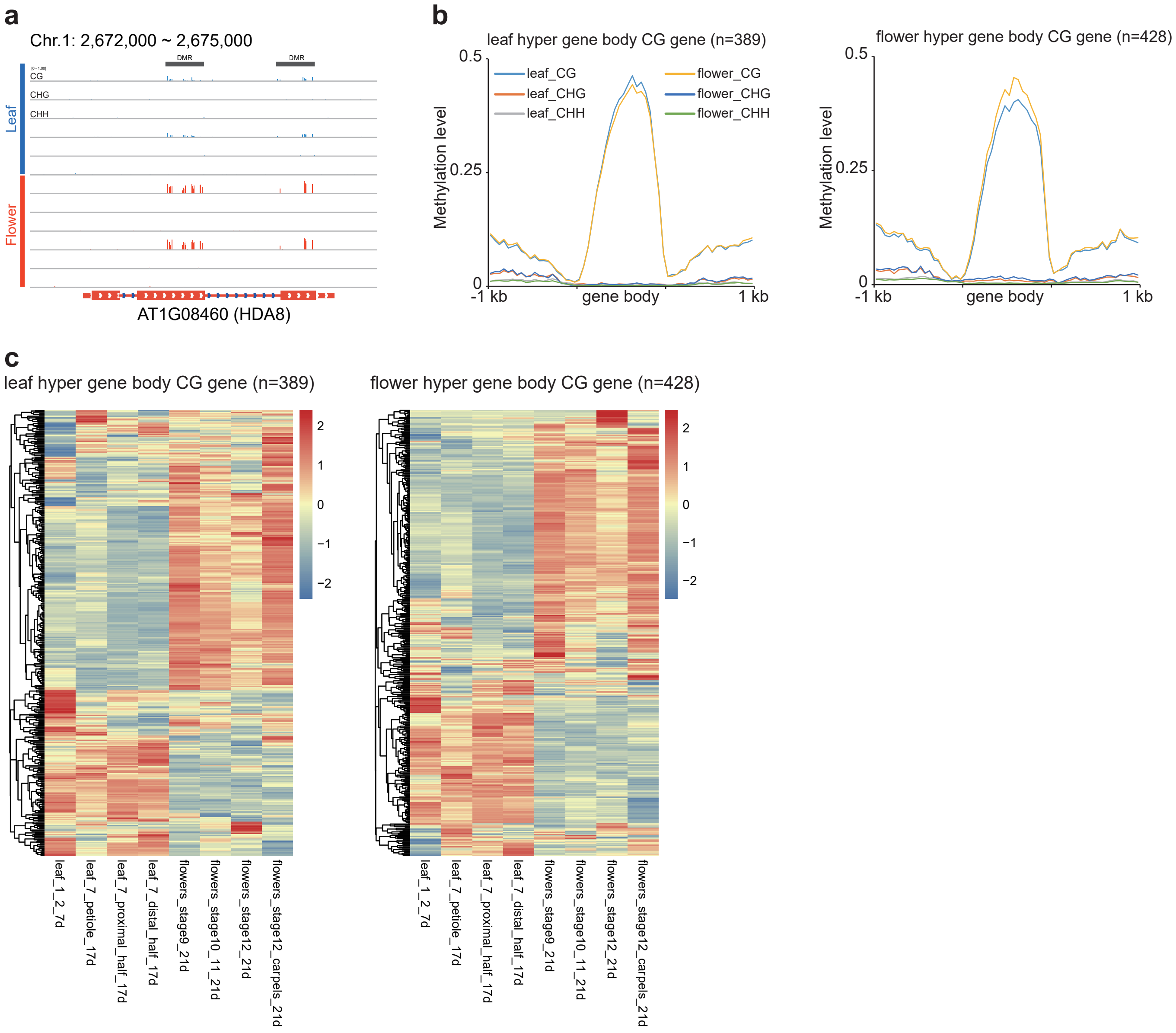

Figure S9 Gene body methylated genes. **a** Genome browser screenshots of flower hyper-CG DMRs in a gene body methylated gene. **b** Metaplots of CG, CHG, and CHH methylations in leaf and flower samples over genes containing hyper-CG DMRs and 1 Kb flanking sequences. **c** Clustering of expression patterns of gene body methylated genes containing hyper-CG DMRs.
